# Evaluation of stability of (1R,2 S)-(−)-2-methylamino-1-phenyl-1-propanol hydrochloride in plasma and urine samples-inoculated with *Escherichia coli* using high-performance liquid chromatography (HPLC)

**DOI:** 10.1007/s11274-024-03890-7

**Published:** 2024-02-13

**Authors:** Khalid S. Almaary, Naiyf S. Alharbi, Rayan M. Al-Huwaymil, Shine Kadaikunnan, Ahmed S. Alobaidi, Jamal M. Khaled

**Affiliations:** Department of Botany and Microbiology, College of Science, King Saud University, P. O. Box 2455, 11451 Riyadh, Saudi Arabia

**Keywords:** *E. Coli*, MPPH, Ephedrine, Forensic investigation, Urine, Plasms, HPLC

## Abstract

The preservation of drug stability in biological evidence during the processes of collection and storage poses a substantial obstacle to the progress of forensic investigations. In conjunction with other constituents, the microorganisms present in the samples play a vital role in this investigation. The present investigation employed the high-performance liquid chromatography (HPLC) technique to assess the stability of (1R,2 S)-(–)-2-methylamino-1-phenyl-1-propanol hydrochloride in plasma and urine samples that were inoculated with *Escherichia coli*. These samples were subjected to storage conditions of 37 °C for 48 h and − 20 °C for a duration of 6 months. Minimal inhibitory concentration (MIC) and Minimal bactericidal concentration (MBC) of MPPH against *E. coli* were determined using microdilution method. The stability of MPPH in plasma and urine samples inoculated with *E. coli* was investigated using HPLC method. The results showed the MIC and MBC of MPPH were 87.5 ± 25 ppm and 175 ± 50 ppm, respectively. While MPPH remained stable in plasma for 48 h at 37 °C, it showed a notable decrease of about 11% in stability when stored in urine for the same period and temperature. From the beginning of the first month, a decrease in the stability of the compound appeared in all samples that were stored at − 20 °C, and the decrease reached 7% for plasma samples and about 11% for urine samples. The decrease in the stability reached its peak in the sixth month, reaching more than 30% and 70% of plasma and urine samples preserved at − 20 °C. This work concluded that *E. coli* can negatively affect the stability of MPPH in plasma and urine samples. This may lead to incorrect conclusions regarding the analysis of biological samples in criminal cases.

## Introduction

Microbial forensics is an interdisciplinary domain that employs microbiology, genetics, and forensic science techniques to examine and assess instances of biological threats, bioterrorism occurrences, outbreaks of infectious diseases, and other criminal or public health issues associated with microorganisms. The primary objective of microbiological forensics is to ascertain and delineate the identity and attributes of microbial agents, discern their sources, and furnish empirical data that can be employed in legal procedures or public health interventions Microorganisms have the potential to function as spatial and temporal physical indicators in several scenarios, notably within the realms of forensic inquiries and environmental surveillance (Budowle et al. 2003; Carter et al. 2017).

The preservation of drug concentrations in biological samples, such as blood and urine, is a crucial determinant in clinical and forensic examinations. Ensuring accurate and dependable results necessitates adherence to proper sample handling and storage conditions. Various elements, such as temperature, time, and the unique features of the drug, can exert an influence on the stability of drug concentrations. According to reports, urine samples are vulnerable to microbial contamination, hence potentially impacting the integrity of medications (Djilali et al. 2022). Ephedrine is a pharmaceutical substance classified as a sympathomimetic amine, frequently employed for therapeutic purposes in the management of several medical ailments. The substance in question is produced from the Ephedra plant and possesses dual characteristics of being a stimulant as well as a decongestant. Ephedrine elicits activation of the sympathetic nervous system, resulting in heightened heart rate, elevated blood pressure, and bronchodilation. It facilitates the secretion of norepinephrine, a neurotransmitter involved in the physiological response known as the “fight or flight” reaction (Morelli and Tognotti 2021). The chemical known as “(1R, 2S)-(–)-ephedrine hydrochloride” can be more precisely described as “(1R,2S)-(–)-2-methylamino-1-phenyl-1-propanol hydrochloride.” The designation (1R,2 S) specifies the stereochemistry of the compound, while the minus sign denotes its optical activity, causing it to rotate plane-polarized light counterclockwise. The substance is characterized by the inclusion of 2-methylamino-1-phenyl-1-propanol as a constituent component, which serves to delineate its chemical structure. The compound in question is propanol, which is composed of a linear chain of three carbon atoms. Specifically, it possesses a methyl group (2-methyl) attached to the second carbon, an amino group (amino) also attached to the second carbon, and a phenyl group (1-phenyl) attached to the third carbon. The inclusion of “hydrochloride” in the compound’s name signifies that it exists in the form of its hydrochloride salt. This method is frequently employed in the pharmaceutical industry to facilitate the preparation of certain medications or chemical compounds for medicinal purposes (Alminshid et al. 2022; Jeber 2019; Zheng et al. 2013).

High-Performance Liquid Chromatography (HPLC) and Gas Chromatography-Mass Spectrometry (GC–MS) are widely employed methodologies in the field of analytical chemistry for the purpose of identifying and measuring ephedrine and its derivatives. The process of identifying ephedrines through the utilization of HPLC involves several sequential stages. These stages encompass the extraction of ephedrine from the given sample matrix, such as blood, urine, or pharmaceutical formulations. Following this, purification and concentration steps are undertaken. Subsequently, a reversed-phase column is employed to effectively separate ephedrine from other constituents presents within the sample. The mobile phase, consisting of a mixture of water and an organic solvent supplemented with an acidic additive, aids in this separation process. Detection of ephedrine is typically achieved through the absorption of UV light, making UV detection, usually at wavelengths around 200–220 nm, a common practice. To ensure accuracy and precision in the analysis, a calibration curve is established, and quantification and quality control samples are employed. These measures serve to validate the reliability of the analysis. In the context of ephedrine detection utilizing GC–MS, the analytical process involves several key stages. Firstly, ephedrine is subjected to derivatization in order to enhance its volatility and stability. Following this, the derivatized ephedrine is extracted from the sample matrix. Subsequently, the ephedrines and their derivatives are separated using a capillary column, employing an appropriate carrier gas such as helium. In the mass spectrometer, the ephedrine molecules are ionized and fragmented. Lastly, a series of standard solutions containing known concentrations of derivatized ephedrine are prepared to establish a calibration curve (Behbahani et al. 2019; Dindar et al. 2020; Eskandarani 2022).

The human body harbors a diverse array of microorganisms, including bacteria, viruses, and fungus, which inhabit various regions of the body and exhibit inter-individual variation (Hamady 2009). Typically, the bloodstream is devoid of microorganisms, and the detection of microorganisms within the bloodstream signifies the existence of an issue that could potentially result in a severe illness and subsequent mortality (Pelletier 1996). *E. coli* is a bacterial species that often resides within the gastrointestinal tract of both humans and animals. Although the majority of *E. coli* strains are innocuous and may even confer digestive benefits, certain strains have the potential to induce sickness. *E. coli* can be categorized into two main groups: commensal strains, which are characterized by their presence in the normal microbial flora, and pathogenic strains, which have the potential to induce illnesses. The commensal *E. coli* plays a crucial role in inhibiting the colonization of the gastrointestinal tract by pathogenic microorganisms, a process commonly referred to as competitive exclusion. Certain strains of Escherichia coli have developed virulence characteristics that enable them to induce infections. Pathogenic strains are commonly classified according to distinct virulence factors and the resultant clinical symptoms they induce. Various types of pathogenic *E. coli* can be identified, including enterotoxigenic *E. coli* (ETEC), which induces traveler’s diarrhea through the production of toxins that impact the intestines; enteropathogenic *E. coli* (EPEC), a prevalent cause of infantile diarrhea in developing nations; enterohemorrhagic *E. coli* (EHEC), responsible for severe foodborne illnesses such as the well-known *E. coli* O157:H7; enteroinvasive *E. coli* (EIEC), which triggers inflammatory diarrhea by invading and destroying intestinal cells; and enteroaggregative *E. coli* (EAEC), associated with persistent diarrhea, particularly among children (Nataro and Kaper, 1998; Russo and Johnson 2000).

The stability of ephedrine and its derivatives in biological samples is affected by the microbial load present, which can originate from external contamination or from the original load in the samples, such as those obtained from individuals with a microbial illness. The underlying hypothesis of this study posits that the presence of bacteria in an individual’s plasma and urine can potentially alter the stability of ephedrine within these biological specimens, hence leading to inaccurate outcomes in chemical analysis. The objective of this study is to assess the stability of ephedrine in plasma and urine samples that have been inoculated with *E. coli* and held at temperatures of 37 and − 20 °C, utilizing HPLC as the analytical technique.

## Methodology

### *Escherichia coli* strain

In this investigation, *Escherichia coli* (Robin) Berkhout 25,922™ was used. The macroscopic characteristics of this strain include that, after one day of incubation on nutrient agar, its colonies become Greyish white colored, convex in height, with a smooth surface, with a translucent-opaque structure. These bacteria cells are typically rod-shaped, with (1–3 μm × 0.4–0.7 μm) (micrometer) in size around 1 μm long, 0.35 μm wide, and 0.6–0.7 μm in volume, single and pairs cells. This bacterium is classified as follows: Bacteria (domain), Bacteria (kingdom), Proteobacteria (phylum), Gamma Proteobacteria (class), Enterobacterales (order), Enterobacteriaceae (family), *Escherichia* (genus), and *Escherichia coli *(*E. coli*) (species).

### Chemical compounds and cultivating media

The compound (1R, 2 S-(–)2-methylamino-1-phenyl-1-propanol hydrochloride, referred to as MPPH, is also known by its (1R, 2 S)-(–)-ephedrine hydrochloride nomenclature. The substance MPPH was acquired from Cerilliant Corporation, a renowned American corporation specializing in the manufacturing of reference chemical compounds used in toxicology and forensic investigation. The Mueller-Hinton broth (MHB), Mueller-Hinton agar (MHA), nutrient broth (NB), nutrient agar (NA), and blood agar base (BA) were procured from Oxoid Ltd., a company based in Basingstoke, UK. The media utilized in this study were made in strict adherence to the directions provided by the manufacturer.

### MPPH-free plasma and urine preparation

Plasma and urine samples were obtained from the Bio House Medical Laboratory (BHML) located in Riyadh, Saudi Arabia. The samples underwent sterilization using a filter membrane with a pore size of 0.22 μm (MF-Millipore®, Sigma-Aldrich). Subsequent chemical analysis verified the absence of any pharmaceuticals, including MPPH, in the samples. The microbiological analysis was conducted to determine the absence of any microorganisms in the samples. The usual plate count method was employed to conduct the test, with incubation carried out under both aerobic and anaerobic conditions at different temperature settings.

### Biological effect of MPPH on *E. Coli* ATCC 25,922

In general, the minimal inhibitory and microbicidal concentrations (MIC and MBC) are used to determine the bioeffect of chemical compounds and natural extracts against selected microbes. In this investigation, a microdilution (two-fold reduction method) was applied to calculate the MIC and MBC of MPPH against *E. coli* ATCC 25,922.

In brief, the stock aqueous solution of MPPH (400 ppm) was prepared using a standard solution of MPPH. The solution was sterilized using MF-Millipore® (0.22 μm pore size). The single pure colony of *E. coli* ATCC 25,922 was obtained using a triple-streak method, and after that, 100 mL of sterile NB were inoculated by a single colony of the tested bacterium. The incubation was done at 36 ± 1 °C for 22 h; after that, 10 mL of the medium were transferred into a new sterile NB, and the incubation was repeated at the same conditions mentioned above. The optical density (O.D) of bacterial growth was 0.65 at 600 nm (it is equivalent 5.5 × 10^6^ CFU/mL). Each well of sterile 96-well polystyrene microplates (Corning®, Sigma-Aldrich) received 90 µL of sterile NB in completely contamination-free working conditions. After that, each well received 10 µL of *E. coli* culture that was prepared as above, except for the wells that were marked as control groups (*E. coli*-free medium). Then, the first well of each row received 100 µL of the aqueous solution of MPPH (400 ppm). After mixing well, 100 µL from the first well was transferred to the next well. The process was repeated in the same manner to the last well in the row, and from there, 100 µL was transferred to an external beaker containing a 70% ethanol solution. The incubation process was conducted under aerobic conditions at a temperature of 37 ± 1 °C for a duration of 24 h. Subsequently, the turbidity measurements were collected for each well for calculating the MICs. The MBC was determined using the sub-cultivation method to figure out the lowest concentration capable of killing all bacterial cells within the specific experimental conditions employed in this study (Sukhikh et al. 2022). The gentamicin was used as a positive control group, and the results showed that the MIC and MBC of this antibiotic were 0.6 and 1.2 µg/mL, respectively. The sterile aqueous solution (sterile distilled water) was used for the negative control group, and the results showed that this solution had no biological activity against the bacteria used in this work.

### Effect of *E. Coli* on stability of MPPH

In vitro experiment was conducted to investigate the impact of *E. coli* ATCC 25,922 on the stability of MPPH in drug-free urine and plasma samples. In this experiment, MIC_50%_ of MPPH was incorporated into the drug-free urine and plasma samples. These samples were previously inoculated with *E. coli* ATCC 25,922 at a concentration of approximately three million cells per milliliter of sample. A portion of the treated samples was incubated at a temperature of 37 ± 1 °C for a duration of 48 h, while the remaining portion was stored at a temperature of − 20 °C for a period of six months. The stability of MPPH was assessed using the HPLC technique at various time intervals, including zero time, 24 h, and 48 h for samples stored at 37 ± 1 °C, as well as zero time and monthly intervals for samples preserved at − 20 °C. For HPLC analysis, the calibration and quantification were done using gradient concentrations of the standard MPPH (0.30, 0.70, 1.50, 3.10, 6.20, 12.50, 25.00, 50.00, 100.00 µg/mL). The column type used in the present work was Agilent -Zorbax-SB-C18, 4.6 mm ID × 150 mm (5 μm), 23 °C, HPLC (Agilent 1290, HPLC, CA, USA). The samples were prepared for analysis following the prescribed procedural steps. During the extraction process, a volume of 1000 µL of methanol (99.9% purity) was combined with 100 µL of the sample. The mixture was then subjected to continuous mixing for a duration of 24 h at a speed of 300 revolutions per minute (rpm) under ambient conditions. The methanol layer was subsequently separated, followed by a repetition of the extraction process for a duration of three hours, conducted five times consecutively. In every stage, the alcohol layers were gathered and combined. The ultimate concentration was attained through the elimination of 90% of methanol. The present study followed the methodology proposed by Jimenez and colleagues, however with several adjustments pertaining to the extraction and preparation of the samples prior to their injection into the HPLC instrument (Jiménez et al. 2006). Control groups were established using samples that were free of microbes and drugs, samples treated just with MPPH, and samples inoculated solely with *E. coli*.

### Design of experiment and statistical analysis

A one-factor completely randomized design was applied to this work; the time was the blocks, and the mean concentrations of MPPH in the samples treated with microbes were calculated during times of different preservation, which were considered the results of the experiment unit. The effect of treatment and time, as well as random error, were analyzed using a one-way ANOVA (OriginPro 2018).

## Results

**Fig. 1:**
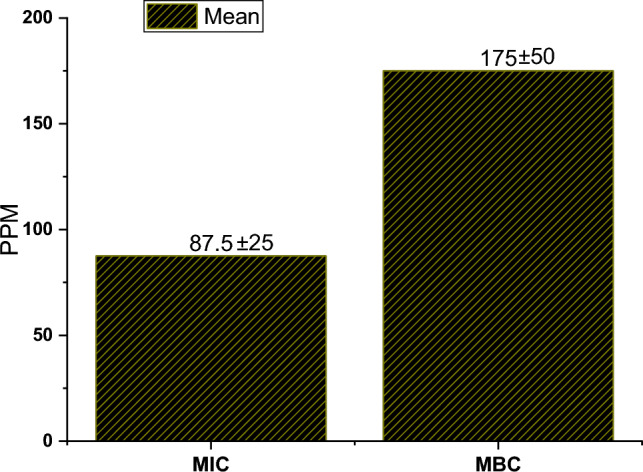
Effect of (1R,2 S)-(–)-2-methylamino-1-phenyl-1-propanol hydrochloride (MPPH) on *Escherichia coli* strain used in this work (*N* = 4). Mean MIC and MBC ± Standard division

The findings and subsequent statistical analysis revealed that MPPH exhibited considerable biological activity against the tested *E. coli* strain in the present study. The MIC and MBC of MPPH were determined to be 87.5 ± 25 and 175 ± 50 ppm, respectively (Fig. 1). The standard data for MPPH in sterile plasma and urine (microbe-free samples) using HPLC showed that retention time (min), width (min), height (mAU*S), and area (%) were 1.632, 0.1148, 56.77945, and 100%, respectively. The results and statistical analysis showed that MPPH had biological activity against *E coli* strain tested in this work.

**Fig. 2:**
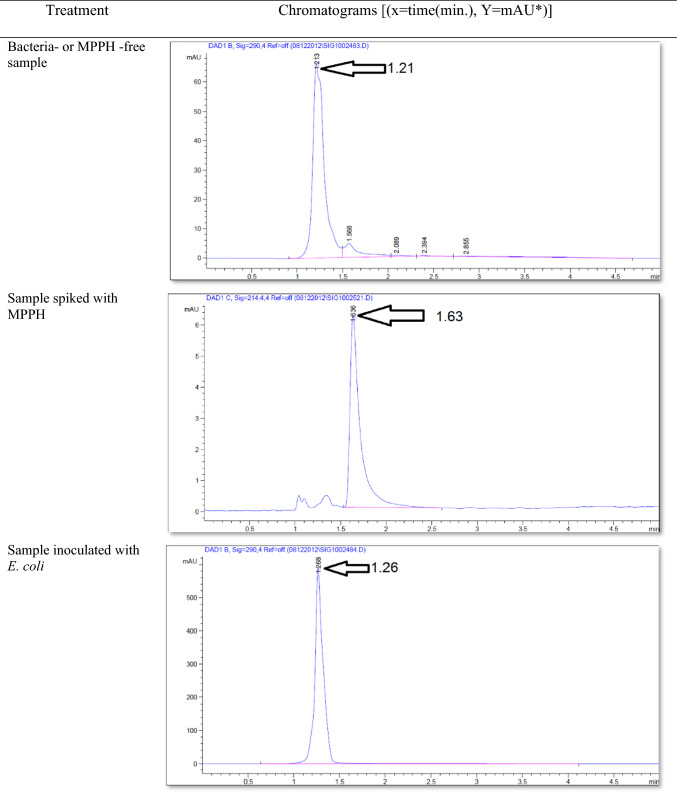

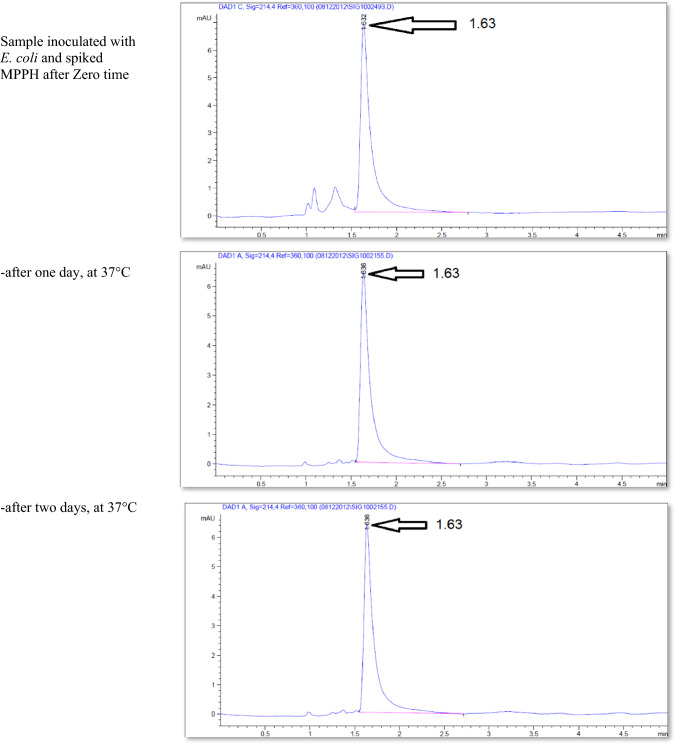

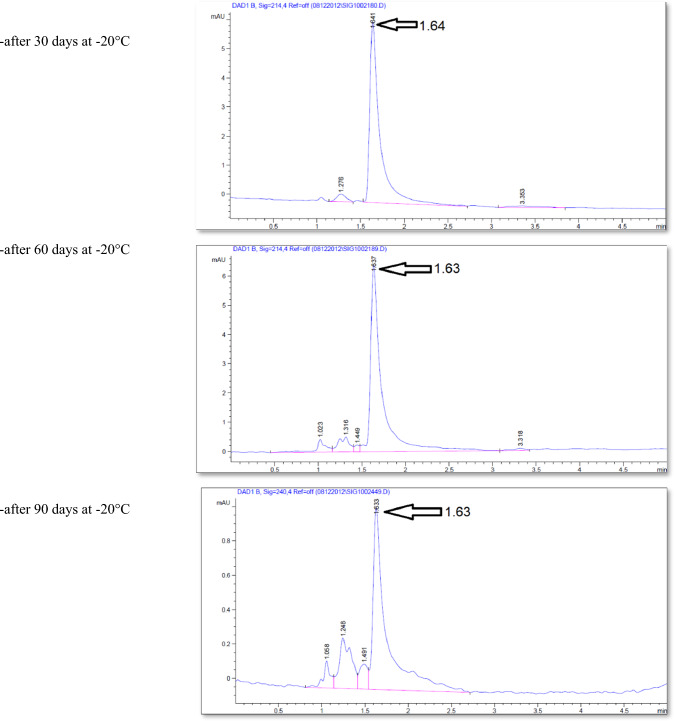

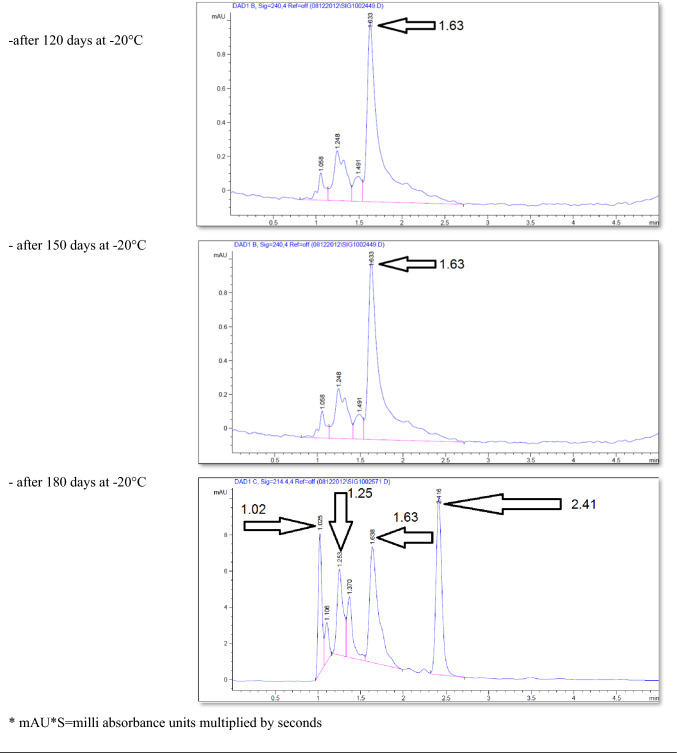
Effect of *Escherichia coli* on stability of (1R,2S)-(–)-2-methylamino-1-phenyl-1-propanol hydrochloride (MPPH) in plasma samples preserved at 37 °C for 48 h, and − 20 °C for 6 months. The chemical analysis was done using HPLC technique at 214.4nm

**Fig. 3:**
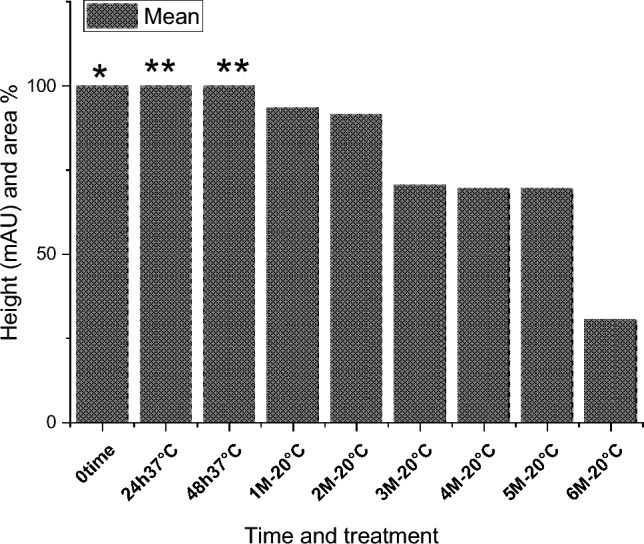
Gradual decrease in stability of (1R,2 S)-(−)-2-methylamino-1-phenyl-1-propanol hydrochloride (MPPH) (%) in plasma samples treated with *Escherichia coli* (*N* = 2). *There are statistically significant differences seen at the 0.05 level when comparing the zero-time group with other groups except 24h 37 °C and 48h 37 °C groups (**). Where 0 time, 24 h 37 °C, 48h 37 °C, 1M—20 °C, 2M—20 °C, 3M—20 °C, 4M—20 °C, 5M—20 °C, 6M—20 °C are Zero time, 24 h at 37 °C, 48 h at 37 °C, 1 month at − 20°C, 2 months at − 20 °C, 3 months at − 20 °C, 4 months at − 20 °C, 5 months at − 20 °C, and 6 months at − 20 °C respectively

The effect of the *E. coli* strain selected in this work on MPPH in plasma samples preserved at 37 °C for 48 h and − 20 °C for 6 months is presented in Fig. 2. The finding showed that time of retention (min.), height (mAU*S), percentage of area (%), and number of peaks for pure MPPH were 1.64, 8.9924, 100%, and one peak, respectively. Regarding the samples free of MPPH and *E. coli*, the height (mAU*S), area (%), and number of peaks were 66.83 and 88.48%, respectively (the retention times of these peaks were 1.21, 1.56, and 2.85 min). The retention time (minutes) of pure MPPH (1.63 min.) was detected in plasma samples that were spiked with MPPH; in contrast, this retention time was not recorded in the other samples to which MPPH was not added. The samples that were stored at a temperature of 37 °C exhibited a retention time of 1.636 min, a height of 6.84616 mAU*S, an area of 100%, and a single peak for a duration of 24 h. Similarly, for a duration of 48 h, the samples displayed a retention time of 1.636 min, a height of 6.9058 mAU*S, an area of 100%, and a single peak. The percentages of MPPH in samples maintained at − 20 °C for durations of 1, 2, 3, 4, 5, and 6 months were found to be 93%, 92%, 70.86%, 70.15%, 70%, and 30.45%, respectively. In relation to the durations in question, the observed number of peaks were as follows: a prominent peak with an area encompassing 93%, another significant peak with an area of 92%, four peaks (with the largest among them covering an area of 70.86%), four peaks (with the largest among them covering an area of 70.15%), three peaks (with the largest among them covering an area of 70%), and six peaks (with the largest among them covering an area of 30.45%), respectively. Figure 3 illustrates the progressive decline in the stability of MPPH (%) within plasma samples subjected to the *E. coli* strain chosen in this study. The statistical analysis revealed that there were statistically significant reductions (*p* < 0.05) observed in all groups as compared to the zero-time group, with the exception of the groups subjected to storage at 37 °C for 24 and 48 h. In these particular groups, MPPH demonstrated perfect stability.

**Fig. 4:**
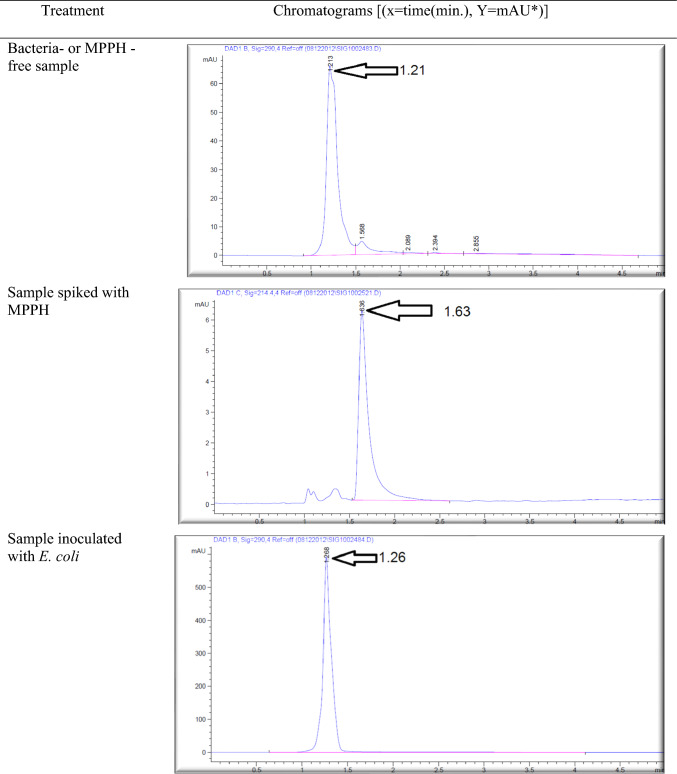

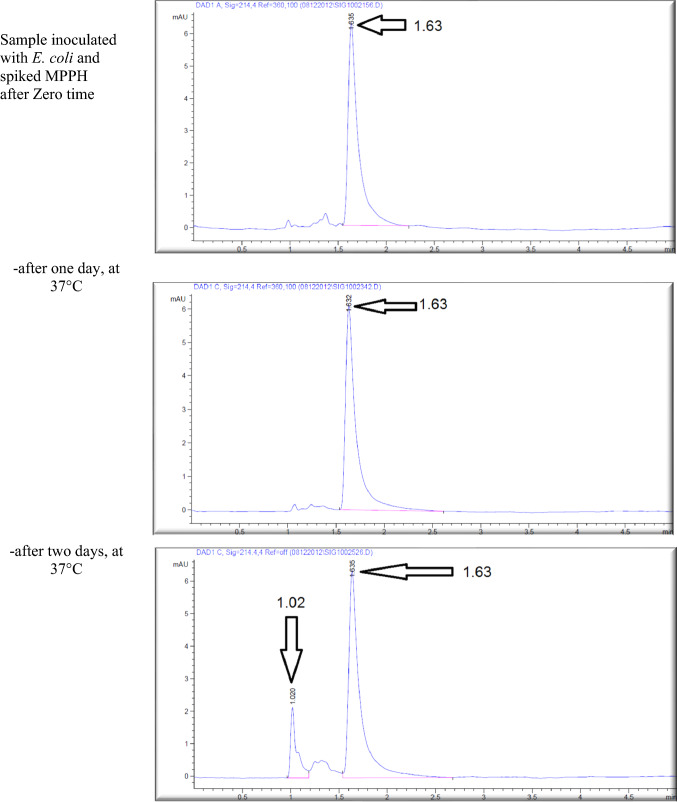

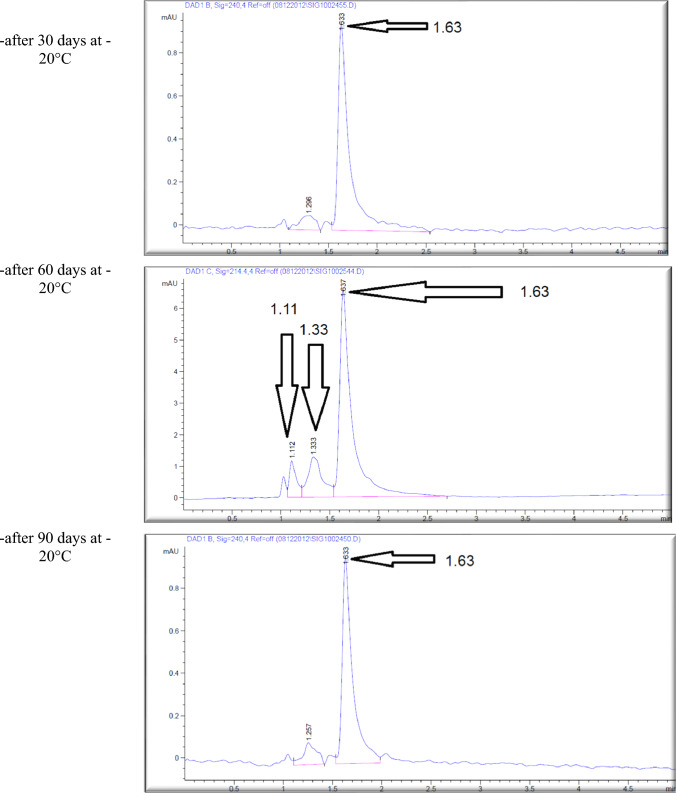

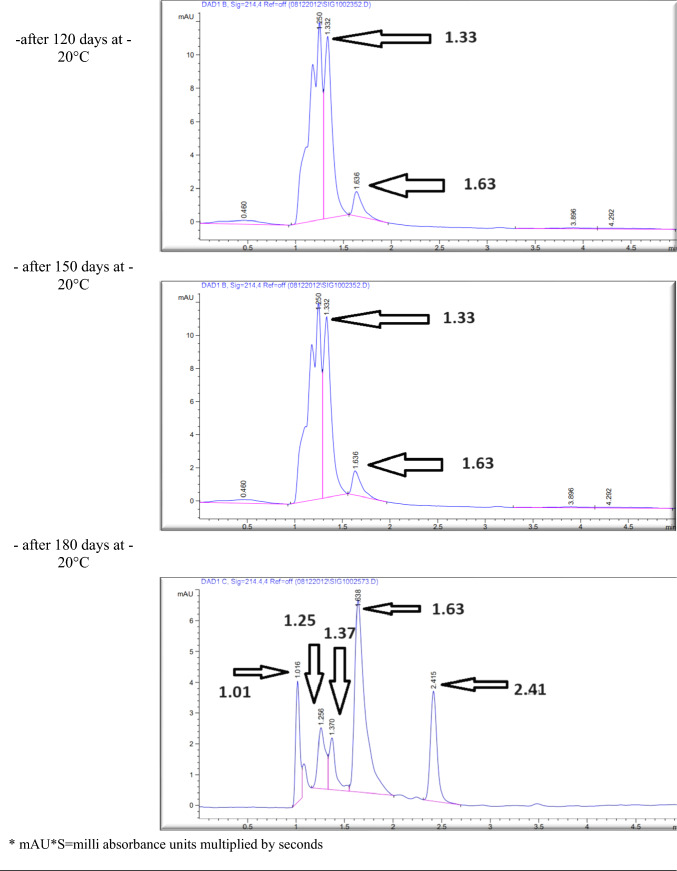
Effect of *Escherichia coli* on stability of (1R,2S)-(−)-2-methylamino-1-phenyl-1-propanol hydrochloride (MPPH) in urine samples preserved at 37 °C for 48 h, and − 20 °C for 6 months. The chemical analysis was done using HPLC technique at 214.4 nm.

**Fig. 5:**
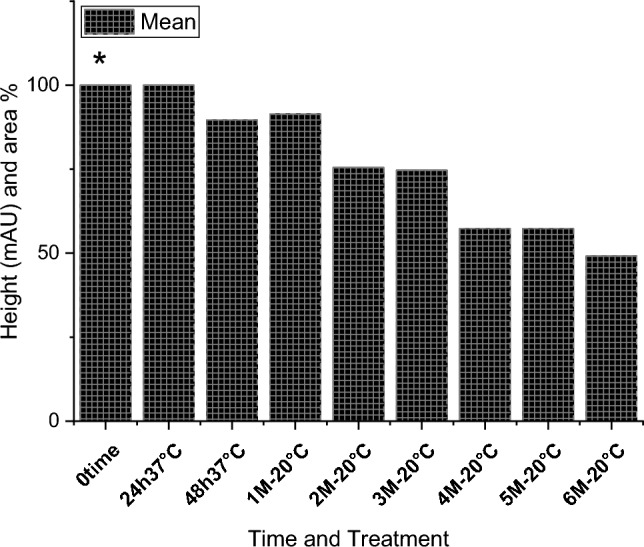
Gradual decrease in stability of (1R,2S)-(−)-2-methylamino-1-phenyl-1-propanol hydrochloride (MPPH) (%) in urine samples treated with Escherichia coli (N=2). *There are statistically significant differences seen at the 0.05 level when comparing the zero-time group with other groups except 24h 37 °C. Where 0time, 24h 37 °C, 48h 37 °C, 1M—20 °C, 2M—20 °C, 3M—20 °C, 4M—20 °C, 5M—20 °C, 6M—20 °C are Zero time, 24 h at 37 °C, 48 h at 37 °C, 1 month at − 20 °C, 2 months at − 20 °C, 3 months at − 20 °C, 4 months at − 20 °C, 5 months at − 20 °C, and 6months at − 20 °C respectively

Figure 4 shows the retention time (min.), height (mAU*S), area (%), and number and nature of peak of MPPH in urine samples inoculated with *E. coli* strain tested in this investigation. The data presented indicates that the MPPH extracted from urine samples exhibited the following values: 1.63 min for retention time, 6.3429 mAU*S for height, and 100% for area. In the urine samples free of MPPH and *E. coli*, three distinct peaks were seen, with corresponding time retentions of 1.21, 1.56, and 2.85 min. The time retention of pure MPPH’s presence was not documented in the aforementioned urine samples and the samples that only inoculated with *E. coli*. With regard to the urine samples that were infected with Escherichia coli (MPPH-free samples), the findings indicated that the retention time (in minutes), height (mAU*S), and area (%) were recorded as 1.26, 5.88670, and 100, respectively. The MPPH was detected in urine inoculated with *E. coli* and spiked MPPH after zero-time groups where retention time (in minutes), height (mAU*S), and area (%) were 1.63, 6.343, and 100 respectively. The compound (MPPH) exhibited stability in the urine samples that were subjected to storage at a temperature of 37 °C for a duration of 24 h. However, it showed a decline in stability exceeding 10% subsequent to a 48 h storage period at the same temperature. The study observed the percentages of MPPH in urine samples stored at a temperature of − 20 °C for different time intervals ranging from 1 to 6 months. The recorded percentages were 91.34%, 75.42%, 74.73%, 57.3%, 57.3%, and 49.16% for the relevant durations. Figure 5 depicts the gradual decrease in the stability of MPPH (percentage) in urine samples that were exposed to the specific *E. coli* strain used for this investigation. The statistical analysis demonstrated that there were statistically significant reductions (*p* < 0.05) found in all groups, except for the group exposed to storage at 37 °C for 24 h when compared to the zero-time group. Within this group, MPPH exhibited remarkable stability.

## Discussion

The presence of microbial contamination, specifically when obtaining samples from individuals afflicted with a microbial infection, is a noteworthy concern within forensic samples. This concern stems from the ability to undermine the integrity of the samples and thus affect the precision of forensic investigations. Forensic samples are commonly gathered at crime scenes and are susceptible to diverse environmental variables that may promote the proliferation of microbes. Continual research and development efforts within the field of forensic science are focused on enhancing methodologies for the identification and mitigation of microbial contamination. Maintaining up-to-date knowledge regarding optimal methodologies for sample collection, storage, and analysis is of utmost significance for forensic scientists and investigators. This is important in order to mitigate the potential adverse effects of microbial contamination on the integrity and reliability of forensic evidence. Furthermore, the ongoing progress in technology and procedures remains vital in enhancing the dependability of forensic analyses. From this perspective, the objective of this study was to establish the impact of *E. coli* in plasma and urine samples on the stability of ephedrine derivatives (MPPH).

According to reports, several strains of *E. coli* are commonly identified in clinical blood and urine samples (Kaper et al. 2004). The research inquiry pertains to the potential of these bacteria to induce alterations in the levels of ephedrine (MPPH) within plasma and urine specimens over varying durations of storage. One notable limitation of our current study revolves around the fact that the previous hypothesis was tested in a laboratory study. These results were supposed to be confirmed in animal experiments. Nevertheless, the current study successfully accomplished its objective of illustrating the detrimental impact of *E. coli* on the stability of MPPH in biological samples subjected to storage conditions of 37 °C and − 20 °C throughout various time intervals.

In this work, the MPPH inhibited *E. coli* at concentrations less than 100 ppm. This finding substantiated the notion that the overuse of ephedrine or its derivatives can have detrimental impacts on the microbial composition of the human body. This finding aligns with a previous study that documented the inhibitory effects of ephedrines on several pathogenic bacteria, including *Streptococcus pneumonia* and *Staphylococcus aureus* (’Bettelheim 1997; Dashtdar et al. 2013).

The preservation and maintenance of biological samples play a critical role in forensic investigations, as they are essential for safeguarding the integrity of evidence and maximizing its utility in legal processes. Biological specimens encompass a range of substances, such as blood, saliva, hair, tissues, and various other biological constituents. The implementation of temperature regulation and the application of appropriate preservatives play crucial roles in forensic investigations, as they serve to inhibit the proliferation of bacteria and the deterioration of DNA or other biological substances. Enzymatic reactions and chemical actions are of paramount importance in the examination of biological materials employed as forensic evidence. These reactions have the potential to yield significant insights into the existence of certain chemicals, facilitate individual identification, and contribute to the reconstruction of events (Skopp 2022). The assay results can be significantly affected by the microbial breakdown of the medicine in biological samples, whether the sample was already contaminated with the microbe or due to external contamination. Morphine glucuronides undergo conversion to free morphine within blood samples, while nitrobenzenes might be modified by intestinal microbes (Carroll et al. 2000).

Preliminary analysis indicates a significant change in MPPH stability within urine and plasma samples stored at − 20 °C. HPLC analysis revealed the emergence of derivative compounds with distinct retention times (min), peak heights (mAU*s), and areas (%). These compounds, potentially arising from MPPH degradation due to bacterial activity (*E. coli* strain used in this study), warrant further investigation. Their precise chemical characterization, metabolic role as potential MPPH metabolites, and the specific enzymatic activities involved in their formation require thorough exploration.

The chemical structure of MPPH exhibits distinct chemical features, including the presence of two chiral centers. The first chiral center, denoted as 1, possesses a R configuration, while the second chiral center, labeled as 2, possesses a S configuration. The presence of a negative sign denotes the compound’s optical activity, signifying its ability to rotate plane-polarized light in a counterclockwise direction. The aforementioned feature is linked to the particular enantiomer of the chemical. The compound under consideration is propanol, which is composed of a linear carbon chain consisting of three carbon atoms. Attached to the second carbon atom is a methyl group, denoted as 2-methyl. Additionally, the second carbon atom is also bonded to an amino group, referred to as amino. Lastly, the third carbon atom is connected to a phenyl group, designated as 1-phenyl. The presence of hydrochloride signifies that the component exists in the hydrochloride salt form, a widely employed method for the pharmaceutical preparation of certain medications or chemical compounds. The E. coli strain used in this work has large enzymatic systems (such as Ala-Phe-Pro-Arylamidase, l-Proline Arylamidase, Ornithine Decarboxylase, Ornithine Decarboxylase, Ornithine Decarboxylase, Succinate Alkalinization, Lysine Decarboxylase, Tyrosine Arylamidase, BETA-Galactosidase and BETA-Glucoronidase) which could play a role in the biomodification of MPPH. Generally, the ephedrine hydrochloride has capacity to both donate and accept hydrogen implies that the observed reactions were of the redox nature, which consequently led to a decline in the concentration of these chemicals in plasma and urine samples including *E. coli*. One illustrative instance is a category of aromatic hydrocarbons wherein a benzene ring, characterized by a six-carbon ring with alternating single and double bonds, is affixed to an alkyl group. The alkyl group, in turn, constitutes a hydrocarbon chain that exhibits variability in terms of both length and structure. This set of compounds is a product of benzene through the substitution of one or more hydrogen atoms with alkyl groups of varying sizes. An alkyl group refers to an alkane molecule that lacks a single hydrogen atom (Lambert 1993).

HPLC is a prevalent analytical methodology employed for the purpose of separating, identifying, and quantifying constituents within a mixture. HPLC is frequently employed as the preferred method for the analysis of ephedrines, a class of alkaloids commonly present in plants such as Ephedra sinica, owing to its high efficiency and sensitivity. There are many studies that have used this method to analyze biological samples to detect ephedrines such as (Abdel Salam et al. 2013; ’Aksu Donmez et al. 2018; Kallinteris et al. 2022). There is a difference between the results of our current study and the study of Kallinteris et al. (2022) (Kallinteris et al. 2022), as we found that the retention time of ephedrines was 1.63 min, while Kallinteris and his colleagues stated that the retention time was 6.31 min. The observed discrepancy could potentially be attributed to variations in the chromatographic parameters employed throughout the experimental procedure, as well as disparities in the composition of the mobile phase.

## Conclusion

The present study has reached the conclusion that MPHH has inhibitory effects against E. coli. Furthermore, it has been observed that the stability of MPHH in urine and plasma samples, which have been inoculated with *E. coli*, is considerably influenced by storage conditions at temperatures of 37 °C and − 20 °C. The MPPH’s stability exhibited a noticeable decline across all samples maintained at − 20 °C, ultimately reaching roughly 30% in the plasma samples after six months and approximately 49% in the urea samples. The decline in stability commenced within the initial month for both types of samples, with MPPH exhibiting a reduction of over 7% in stability in plasma samples and roughly 9% in stability in urine samples. The findings of this study correspond with the previous works, which consistently highlight the significant risk posed by microbial contamination to the integrity and reliability of biological samples utilized in forensic investigations. Furthermore, the acquisition of samples from individuals afflicted with bacterial diseases may affect the results of the investigation and cause the samples to deteriorate during long-term storage.

## Data Availability

All raw data used in this manuscript are available from the corresponding author.
